# Simultaneous antegrade and retrograde endoscopic treatment of non-malignant ureterointestinal anastomotic strictures following urinary diversion

**DOI:** 10.1186/s12894-017-0252-0

**Published:** 2017-08-08

**Authors:** Weiguo Hu, Boxing Su, Bo Xiao, Xin Zhang, Song Chen, Yuzhe Tang, Yubao Liu, Meng Fu, Jianxing Li

**Affiliations:** Department of Urology, Beijing Tsinghua Changgung Hospital, Tsinghua University, No. 168 Litang Road, Changping District, Beijing, 102218 China

**Keywords:** Ureterointestinal anastomotic stricture, Antegrade, Retrograde, Endourology, Urinary diversion

## Abstract

**Background:**

The ureterointestinal anastomosis stricture (UAS) is a common complication of urinary diversion after radical cystectomy. For decades, open anastomotic revision remained the gold standard for the treatment of UAS. However, with the advancement in endoscopic technology, mini-invasive therapeutic approaches have been used in its management. Here, we report our experience with and long-term results of combined simultaneous antegrade and retrograde endoscopy (SARE) in the treatment of non-malignant UASs after urinary diversion in a consecutive series of patients.

**Methods:**

From March 2012 to January 2015, there were 32 consecutive patients with 32 non-malignant UASs following radical cystectomy and urinary diversion. Twenty-nine patients were treated with SARE technique and comprised the study group. Using simultaneous antegrade flexible ureteroscope combined with retrograde semi-rigid ureteroscope or nephroscope, partial or complete strictures were managed with laser incision and balloon dilation under direct visualization. A 7/12 Fr graded endopyelotomy stent was left for 3–6 months after the procedure. Success was defined as symptomatic improvement and radiographic resolution of obstruction.

**Results:**

With a median followup of 22 months (6–36), the overall success rate for SARE was 69.0%. Twenty patients with partial stricture had a success rate of 85%, and 9 patients with complete stricture had a success rate of 33.3%. Renal function, hydronephrosis grade, stricture type, and stricture length were significant influences on the outcome (*P* < 0.05). No complication was observed.

**Conclusions:**

The SARE is a safe and effective treatment for UAS, and may be the only endoscopic treatment approach for complete UAS. While success rate for complete strictures is low compared to open revision, it should be considered as an initial approach given its low overall morbidity. For partial strictures, prudent patient selection results in higher success rates that are nearly comparable to open revision.

## Background

Despite the advances and modifications in urinary diversion after radical cystectomy, the ureterointestinal anastomosis stricture (UAS) is still a common complication of this procedure. The reported incidence of UAS following urinary diversion ranges from 3 to 10% [1, 2], depending on clinical factors such as patient series, intestinal segment and anastomosis type [3]. Standard management of UAS involves open surgical revision of the anastomosis with reimplantation of the viable ureter into the urinary diversion. Despite a high success rate of greater than 80%, open anastomotic revision is often a technically challenging procedure, leading to considerable morbidity and prolonged hospitalization [4].

In the recent decades, treatment for UAS has changed drastically as a result of advances in endoscopic techniques and instrumentation [5]. Various endoscopic methods have been employed to treat UAS, such as balloon dilation [2], or endoureterotomy using cold-knife [6], electrocautery [7] or laser [8]. However, in most previously published studies, endourologic treatment of ureterointestinal strictures have employed either an antegrade or retrograde access exclusively. It is always challenging to identify the ureterointestinal anastomosis in the retrograde approach. The antegrade approach has been limited to treating only partial UAS.

In the current series, we describe a surgical endoscopic technique performed by adopting a simultaneous percutaneous antegrade flexible ureteroscope combined with retrograde semi-rigid ureteroscope or nephroscope. We retrospectively reviewed 29 partial and complete nonmalignant UASs treated with this technique to evaluate its efficacy and safety during the long-term followup.

## Methods

From March 2012 to January 2015, there were 32 consecutive patients (19 males and 13 females) with 32 non-malignant UASs following radical cystectomy and urinary diversion. Of these patients 3 with complete stricture longer than 2 cm were excluded from analysis. Open surgery had to be performed on these patients. The remaining 29 people were treated with combined simultaneous antegrade and retrograde endoscopy (SARE) technique and comprised the study group. The mean age was 55.7 years (range 39–73 years). The indications for these urinary diversions were transitional cell carcinoma of the bladder. Patients with extrinsic ureteral compression, or tumor at the anastomotic site were not included.

The main symptoms were flank pain (*n* = 8) and urinary infection (*n* = 4). Fourteen patients were asymptomatic at presentation. Three patients presented with biochemical and/or clinical evidence of renal failure. Of all the patients, 20 had partial strictures and the remaining 9 had complete strictures. Twenty-six had a Bricker urinary diversion and 3 had a Studer orthotopic neobladder. Four patients received initial drainage with a nephrostomy tube, and antibiotics were used in 9 patients prior to definitive treatment, other patients were treated initially with this combined approach after the UAS were diagnosised. The mean interval between the urinary diversion and the initial diagnosis of UAS formation was 22 months (ranged 3–51). In all patients, UAS was diagnosed by computerized tomography urography (CTU) and/or antegrade pyelography. Complete stricture was diagnosised if the contrast can’t pass the strictured anastomosis. Abdominal CT was performed preoperatively in all patients to exclude tumor metastasis or local recurrence. Neobladder cystoscopy was performed to rule out urethral or neobladder neck stricture or tumor recurrence. When poor renal function was suspected on radiographic imaging, renal scan was performed. Stricture site, length and degree of patency were obtained from the preoperative adjunct imaging studies or the operative notes. Biopsy of the strictured anastomosis area was obtained when necessary. Preoperative hydronephrosis was found in all patients by sonographical and radiological studies. Hydronephrosis was graded asI- mild pelvic dilatation only, grade II- moderate caliceal dilatation, grade III- severe caliceal dilatation and grade IV- caliceal dilatation with renal parenchymal atrophy.

Under general anesthesia, the patients with Bricker bladders were placed in a modified oblique supine position with raised (approximately 20–30°) nephrostomy side. Patients with orthotopic neobladders were placed in the modified lithotomy position with access to the flank region for nephrostomy. Optimal percutaneous access was performed under ultrasonographic guidance using the two-step method as we mentioned previously [9].

After percutaneous access was achieved, concurrent renal or ureteral calculi were removed firstly. Then, an 8.5–9 Fr flexible ureteroscope was passed antegrade over a guidewire to the strictured area. Under direct vision, the guidewire was passed through the stricture down to the pouch. If the anastomosis was obstructed completely, the modified cut-to-the-light technique was employed. Briefly, we turned off the light of the flexible ureteroscope, and performed the incision towards another illuminated endoscope placed retrograde to the distal end of the stricture using a 200 μm holmium: YAG laser fiber. The incision was usually made in the depression of the mucosa located in proximal end of the stricture. When the stiff end of the 0.032-in. hydrophilic guidewire (Bard Medical Division) passed through the stenotic segments, it was extracted ureteroscopically or nephroscopically from the intestinal urinary pouch with a grasp to get through-and-through access. The X Force® U30 balloon dilator (Bard Medical Division) was then placed in UAS segment in retrograde way and inflated under direct visual guidance. We typically dilated the balloon to 21 Fr (25 atm) and left it inflated for 5 min. Endoureterotomy was performed following balloon dilation using the 200 μm, end firing, pulsed, 80 W holmium: YAG laser (energy 0.6–2.0 J and rate 10–15 Hz) under direct vision.

Pulsations at the stricture area were evaluated prior to the endoureterotomy. The incision was made anteriorly over the iliac vessels, anteromedially over the internal iliac vessels to avoid vascular injury. A full-thickness incision was made into the periureteral adipose tissue, extending about 5 mm above and below the strictured segment. In severe strictures, the stenotic segments can be pre-dilated with fascial dilators before retrograde balloon dilation.

A 7/12 Fr graded endopyelotomy stent (Urovision, Germany) was left in situ for 3–6 months postoperatively. Routine followup consisted of history, physical examination and renal ultrasound every 3 months in year 1 and biannually thereafter. CT and/or diuretic renography were performed if necessary. Success was defined as radiographic resolution of obstruction and symptomatic improvement without the need for ureteral stents or nephrostomy tubes.

Time to the last followup in successfully treated patients was considered a censor point and time to failure was considered as end points for assessment using Kaplan-Meier analysis. Associations between different clinicopathological factors and success were analyzed to predict the outcome, using Student t or Wilcoxon rank sum test t for continuous data and the standard chi-square or Fisher’s exact test for categorical data with *p* <0.05 considered statistically significant.

## Results

**Fig. 1:**
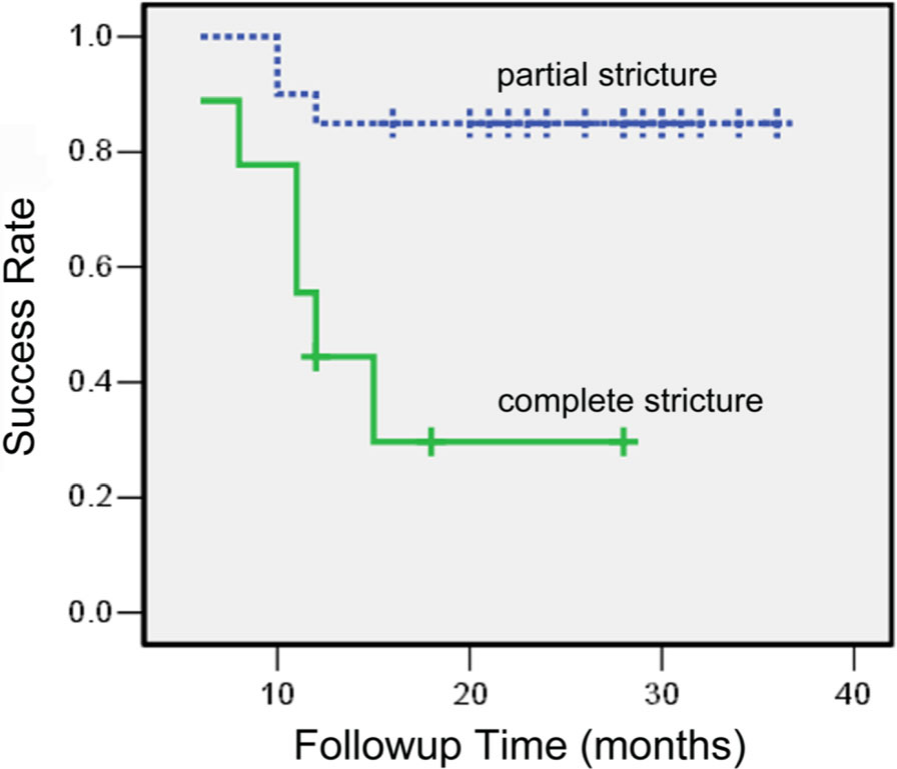
Kaplan-Meier curve of success rate with time of combined simultaneous antegrade and retrograde endoscopic treatment for partial and complete stricture of ureterointestinal anastomosis. Cross hatches indicate censored cases with no obstruction at last follow-up

The median followup time was 22 months (range 6–36), and the overall success rate was 69.0% (20 of 29 UASs). No serious perioperative complications or urinary tract infections were noted. With a median followup of 27 months (range 10–36) for patients with partial strictures, the success rate was 85% (17 out of 20). For the patients with complete strictures, after a median followup of 12 months (range 6–28), the success rate was 33.3% (3 out of 9). Figure 1 shows a Kaplan-Meier curve of success rate of SARE treatment for the complete and partial UAS respectively. The average operation time was 26 min (ranged 15–60), with minimal blood loss. The average hospital stay was 3.7 days (ranged 3–5). In the 9 failed patients, restenosis occurred 5.6 (range 1–9) months after the removal of the ureteral stent. Failure was managed by open anastomotic revision in 4, permanent indwelling stent drainage in 2 and nephrostomy in 3. Table 1 lists the categorical data for patient and stricture characteristics, and the success rates are presented accordingly. The UAS located in 16 ureterorenal units (URU) on the left side and 13 on the right. Nine of the patients had stricture associated with ureteral calculi. While kidney function, hydronephrosis grade, and stricture type significantly influence the results of the treatment (*p*<0.05=, side of the stricture, history of endoscopic therapy, co-existence of ureteral calculi or urinary diversion type seem to be independent of the outcome of endourological treatment (*p*>0.05). We didn’t find a statistically significant association between prior radiation and outcome, this maybe due to limited number of patients (only 2 with radiation history) included in our analysis. Table 2 shows the continuous data for patient and stricture characteristics. Our analysis suggested that the age of the patients, postoperative stent duration (3–6 months) or the period to the diagnosis of UAS after original conduit creation had no influence on the outcome. However, our data demonstrated that the stricture length was significantly associated with the prognosis of outcome.

**Table 1:** Statistical analysis of categorical data for patient and stricture characteristics (*n*=29)

Characteristics	No. Successes /No. Patients (%)	*P* ^a^ value
Gender:		0.454
Male	10/16 (62.5)	
Female	10/13 (61.5)	
Side:		1.000
Left	11/16 (68.8)	
Right	9/13 (69.2)	
Irradiation:		0.089
Yes	0/2 (0)	
No	20/27 (74.1)	
% Preop ipsilateral renal function:		0.002
≥25	17/19 (89.5)	
<25	3/10 (9.1)	
Hydronephrosis grade:		0.003
I―II	15/16 (93.8)	
III―IV	5/13 (38.5)	
Diversion type:		1.000
Studer orthotopic neobladder	2/3 (66.7)	
Bricker	18/26 (69.2)	
Stricture type:		0.010
Complete	3/9 (33.3)	
Partial	17/20 (85.0)	
Past therapy:		0.287
Endoscopic	2/5 (40)	
None	18/24(75.0)	
Co-existence of ureteric calculi:		0.088
Yes	4/9 (44.4)	
No	16/20 (80.0)	

^a^Fisher’s exact test

**Table 2:** Statistical analysis of continuous data for patient and stricture characteristics (*n*=29)

Variable	Total	Success	Failure	*P* Value
Age (yr)	55.72 (8.61)	56.45 (8.48)	54.11 (9.19)	0.508^a^
Interval to stricture formation (mo)	21.52 (11.06)	21.10 (9.84)	22.44 (14.04)	0.768^a^
Stricture length (cm)	1.29(0.34)	1.12 (0.25)	1.67 (0.14)	0.0001^a^
Stent duration (mo)	4.66 (1.47)	4.50 (1.54)	5.00 (2.32)	0.501^b^

Data presented as the mean, with the standard deviation in parentheses

^a^Student’s t test

^b^Wilcoxon rank sum test

## Discussion

The non-malignant UAS can be caused by anastomotic technique associated ischemia, avascular necrosis or perianastomotic fibrosis due to chronic inflammation, edema or urine leakage [10]. For many decades, open surgical revision of the anastomosis remained the gold standard for the management of ureterointesitinal stricture with an 80–91% reported success rate [4, 11]. However, the open surgical procedures can be difficult to perform, and associated with considerable morbidity, due to dense adhesions or fibrosis caused by previous surgery or radiotherapy. The advancement in urologic endoscopic technology has facilitated minimally invasive therapeutic approaches for the treatment of ureterointesitinal stricture, leading to less complications and shorter convalescence time. Although there were plenty of reports describing different endourological techniques for treatment of UAS, most of them employed either an antegrade or retrograde approach alone and currently there is no report suggests that any modality is superior to another (Table 3).

**Table 3:** Endoscopic management of non-malignant ureterointestinal anastomotic strictures

Technique	Study	Procedures	Approach^a^	Mean follow-up (mo)	Success rate (%)
Balloon dilation	Nassar et al. [3]	16	A	43	50
	Yagi et al. [19]	13	A	47.1	77
	Ravery et al. [17]	14	A	16	61
	DiMarco et al. [2]	52	A/R	24	15
	Kwak et al. [20]	18	A	6	28
Laser incision	Mihoua et al. [18]	15	A	11.5	33
	Laven et al. [4]	16	A	35	50
	Watterson et al. [8]	24	A	22.5	70.8
Cold-knife incision	Nassar et al. [3]	21	A	43	52.3
	Poulakis et al. [6]	43	Combined	38.8	60.5
	Poulakis et al. [7]	22	A	23.5	74
Electrocautery incision	Lovaco et al. [5]	25	Combined	51	80
	Meretyk et al. [21]	14	R	28.6	57
Acucise cutting balloon device	Cornud et al. [22]	37	A	25	67.5
	Lin et al. [23]	10	A	13	32
	Babayan et al. [24]	9	A	3	33
Multiple modalities	Wolf et al. [16]	30	A	13	32

^a^ *A* antegrade, *R* retrograde, *A/R* antegrade or retrograde

The concept of combined simultaneous antegrade and retrograde endoscopic approach offers several advantages over either modality used alone. Firstly, in the SARE approach, antegrade placement of the flexible ureteroscope permits cooperative treatment of the stenotic lesion with retrograde modality over through-and-through guidewire, which provided the control required to ensure full-thickness and full-length stricture incision under direct visualization. The 85% success rate nearly comparable to open revision in patients with partial strictures in our analysis further proved that the SARE is an effective treatment approach. Secondly, the SARE permits the using of “cut-to-the-light” technique to get the through-and-through access, which may be the only way to endoscopically treat the complete obstruction. In our patient series, the success rate for complete obstruction is 33%. Long-term and large-scale studies are needed to further explore this method. However, given its low morbidity compared with open revision, the SARE treatment should be considered initially in patients with complete strictures.

Cut-to-the-light technique had been described to establish through and through access in complete obliteration of ureteral strictures [12]. However, few studies have been done to evaluate the long term results of this procedure. Goda et al. [13] described a case of complete ureteral stricture managed by endoscopic recanalization using the cut-to-the-light technique through potassium titanyl phosphate (KTP) laser ureterotomy. No signs of restenosis were observed 24 months after endoscopic treatment. In our series, this technique was successfully performed in 9 patients with complete strictures shorter than 2 cm, and 3 complete UASs remain patent after a median followup of 12 months. To our knowledge this is the first series of patients with complete UAS treated by this modified technique with long term followup.

Our analysis shows that factors associated with success rate are renal function, hydronephrosis grade, stricture type, and stricture length. Decreased ipsilateral renal function has been reported in several studies as a risk factor for failure of endoscopic treatments of the ureteral stricture disease [14, 15]. Wolf et al. found that no patient with renal function less than 25% had successful endoureterotomy in a series of 47 ureteral strictures [16]. Poulakis et al. reported that all the patients who failed in treatment with cold knife incision had less than 25% ipsilateral renal function in a series of 22 UASs [7]. In our series, 11 patients had less than 25% renal function and treatment failed in 10. Few groups have examined the impact of hydronephrosis on the success rate of endoscopic treatment of non-malignant UAS. Our study shows that significant hydronephrosis predicts a higher failure rate. 94.4% of UAS were successfully treated in patients with renal hydronephrosis leveled gradeI―IIverse 21.4% in patients leveled grade III―IV.Similar results were also reported by Poulakis and his colleagues in a series of 40 patients with 43 UASs underwent cold-knife endoureterotomy [6].

Several studies found ureteral stricture length to have statistically significant influence on the result of endourological intervention with decreasing ureteroscopic success rate as the stricture length increases [15]. One series of 18 patients with 22 non-malignant UASs who underwent antegrade cold-knife endoureterotomy found that 72.7% of the patients with strictures>1.5 cm failed the endoscopic management, while all patients with stricture lengths ≤1.5 cm succeed [7]. In our analysis, the length of the stenotic portion of the ureterointestinal anastomosis ranged from 0.5 to 1.8 cm, and our results showed shorter stricture length strongly correlated with higher success rates (Table 2).

Regarding postoperative stenting, a stent duration of 6–8 weeks is widely accepted in many published studies [7], however, there is no large scale randomized clinical trial published to date demonstrating the optimum stent duration after endoureterotomy. Ravery et al. postulated that the prolonged ureteral stenting might have promoted ureteric healing and attributed the high success rate of 61% to the increased duration of stenting (4–30 months) [17]. Wolf et al. proved statistically that the stent duration (≤4 Vs.>4 weeks) did not influence the short- and long-term success rate of endoureterotomy in cases of both benign ureteral strictures and ureteroenteric ones [16]. In our analysis, we found that prolonged stent duration (range 3–6 months) had no beneficial effects on the clinical outcome.

Our study has several limitations. First, it is limited by its retrospective and single-institution study design. Future prospective, large-scale and long-term studies are needed in multiply centers. Secondly, the stent duration in our series is 3–6 months postoperatively based on our experiences, however, our data showed that prolonged stent duration was not significantly associated with high success rate, further study should be proposed to find the optimum stent duration and its correlation with clinical outcome. Thirdly, our study can’t explain the influence of failed balloon dilation and laser incision, especially for complete UAS obstruction, on the success rate of subsequent open surgical revision. However, the previously published data have showed that there was no statistically significant difference in open surgical revision outcomes for patients with and without prior endoureterotomy [4, 18]. Fourthly, due to the small cohort size and limited number of events, we were unable to perform multivariate analyses to identify independent predictors of success. Further study is needed to explore this.

## Conclusions

Conventional endourologic interventions for UAS, such as antegrade balloon dilation, have lower success rates and have been limited to treating only partial strictures. Our study is the first series of patients with partial and complete UASs treated with endoscopic method. Although the success rates of SARE for complete strictures are low compared to open revision, it should be considered initially as it may be the only endoscopic treatment approach for complete strictures. For incomplete strictures, selection of patients with the most favorable prognostic factors, such as better renal fuction, lower hydronephrosis grade and shorter stricture length, will lead to excellent success rates nearly comparable to open revision. Further perspective studies with more patients and longer followups are needed in order to validate our conclusion regarding the practice of SARE in patients with non-malignant UASs after urinary diversion.

## Abbreviations

CTU: Computerized tomography urography
SARE: Simultaneous antegrade and retrograde endoscopy
UAS: Ureterointestinal anastomotic stricture
URU: Ureterorenal units
